# Association Between 24‐h Movement Behavior, Physical Fitness, and Inhibitory Control in School Adolescents: A Complex Network Analysis

**DOI:** 10.1002/ajhb.70082

**Published:** 2025-06-06

**Authors:** Rafael Santos dos Cruz, Ana Clara Cassimiro Nunes, João Paulo Rodrigues dos Santos, Vagner Deuel de O. Tavares, Isabela Almeida Ramos, André Igor Fonteles, Paulo Felipe Ribeiro Bandeira, Clarice Maria Lucena de Martins, Rodrigo Alberto Vieira Browne

**Affiliations:** ^1^ Graduate Program in Physical Education Catholic University of Brasília Brasília Federal District Brazil; ^2^ Graduate Program in Physical Education Federal University of Vale do São Francisco Petrolina Pernambuco Brazil; ^3^ Faculty of Nursing University of Calgary Calgary Alberta Canada; ^4^ Regional University of Cariri Department of Physical Education Crato Ceará Brazil; ^5^ Faculty of Sports University of Porto Porto Portugal

**Keywords:** adolescence, cognitive function, health risk behaviors, physical activity, systems theory

## Abstract

**Purpose:**

To investigate the interrelationships between 24‐h movement behaviors, health‐related physical fitness, and inhibitory control performance in adolescents.

**Methods:**

This cross‐sectional study included 216 Brazilian adolescents (aged 16.7 ± 1.2 years) from a federal public school. Movement behaviors—moderate‐to‐vigorous physical activity (MVPA), smartphone screen time, sleep duration, and excessive daytime sleepiness—were assessed using the Global School‐based Student Health Survey, digital well‐being tools, and the Pediatric Daytime Sleepiness Scale. Aerobic capacity was measured using the PACER test, muscular strength by the FitnessGram push‐up test, and body composition through body mass index. Inhibitory control was assessed using the Flanker task (E‐Prime v3.0). Separate network analyses were performed for congruent and incongruent reaction times (RT).

**Results:**

Physically active adolescents had faster RTs than their insufficiently active peers, with physical activity negatively associated with RT in both the congruent (−0.116) and incongruent (−0.125) networks. Aerobic capacity (e.g., expected influence: 0.879–0.902) and muscular strength (expected influence: 1.360–1.384) appeared as central components in both network structures. However, no associations were found between sleep duration, screen time, or excessive daytime sleepiness and inhibitory control.

**Conclusions:**

Adherence to MVPA guidelines was directly associated with improved inhibitory control performance among adolescents. Health‐related physical fitness, particularly aerobic capacity and muscular strength, was indirectly associated with inhibitory control. Other movement behaviors were not associated with cognitive performance in this sample.

## 1 Introduction

Adolescence is a stage marked by biological, psychological, and social changes that can result in both adaptive explorations and risk behaviors (Bozzini et al. 2021; Walsh and Nicholson 2022). In this period, neurocognitive development is crucial for executive function (EF), which is responsible for managing thoughts, actions, and emotions (Nelson et al. 2019). Inhibitory control is a significant component of EF, involving the ability to suppress habitual impulses and maintain focus on goals (Diamond 2020). Inhibitory control performance is associated with better academic achievement and an increased capacity for learning (Privitera et al. 2023). Conversely, impaired inhibitory control is linked to the consumption of harmful substances, including drugs and alcohol (Herman and Duka 2020), as well as excessive digital media consumption (Shoshani et al. 2024). Additionally, movement‐related behaviors may be connected to inhibitory control performance, particularly during adolescence (de Greeff et al. 2018).

Movement behaviors over 24 h include physical activity, sedentary behavior, and sleep (Tremblay et al. 2016). Interestingly, studies have demonstrated that adherence to healthy movement behavior guidelines is associated with improved cardiometabolic and mental health (Howie et al. 2023; Sampasa‐Kanyinga et al. 2020), cognitive functioning (Brush et al. 2024), health‐related physical fitness, such as aerobic capacity and muscle strength (Higgins et al. 2020; Tapia‐Serrano et al. 2023), and reduced risk of obesity (Howie et al. 2023). Additionally, aerobic capacity has been positively associated with inhibitory control performance, mediated by body mass index in the adolescent population (Cabral et al. 2021). Felin Fochesatto et al. (2023) investigated the interconnections of 24‐h movement behaviors, cognitive function, mental difficulties, and physical fitness in 226 children aged 6 to 11 years using a complex networks approach. An association was found between moderate‐to‐vigorous physical activity and screen time with cognitive function, as well as the importance of physical fitness in the topology of the complex network, highlighting it as a key intervention variable.

A robust body of evidence has highlighted the interrelationship between 24‐h movement behaviors, health‐related physical fitness, and cognitive function (Fung et al. 2023; Howie et al. 2023). However, the few studies that have employed a complex network approach to investigate these associations have predominantly focused on children (de Martins et al. 2020; Felin Fochesatto et al. 2023), leaving a gap in understanding how these variables interrelate in the adolescent population. Moreover, most of the existing research has been conducted in high‐income countries, limiting the generalizability of findings to diverse socioeconomic and cultural contexts (Fung et al. 2023; Howie et al. 2023; Wu et al. 2011). In Brazil, significant disparities in 24‐h movement behaviors are influenced by regional, environmental, and socioeconomic factors (C. Martins et al. 2024). These disparities highlight the need for studies in Global South countries, where structural and contextual influences on adolescent behavior may differ. The complex network approach aids researchers in understanding the simultaneous nonlinear interactions between variables and how they self‐organize to produce an effect, offering insights into representing and measuring complex systems (Felin Fochesatto et al. 2023; de Martins et al. 2020). Thus, this statistical choice provides an innovative perspective compared to traditional linear methods such as regression analysis, correlations, and structural equation models (Leme et al. 2020).

By analyzing these interrelationships in a Brazilian adolescent sample, this study offers new perspectives on how 24‐h movement behaviors, physical fitness, and inhibitory control interact in a population characterized by socioeconomic and environmental heterogeneity. Therefore, the objective was to explore the interrelationships between 24‐h movement behaviors, health‐related physical fitness, and inhibitory control performance in school‐based adolescents using the complex network approach.

## 2 Methods

### 2.1 Design Study

This cross‐sectional study was conducted at a federal public school in Sousa, Paraíba, Brazil, from June to October 2023. The research followed the ethical principles outlined in the Declaration of Helsinki and the Brazilian National Health Council Resolution No. 466/2012 and was approved by the Research Ethics Committee (CAAE No. 49857421.0.0000.5184). The study also adhered to the STROBE guidelines for reporting observational studies (Malta et al. 2010).

### 2.2 Participants

**FIGURE 1:**
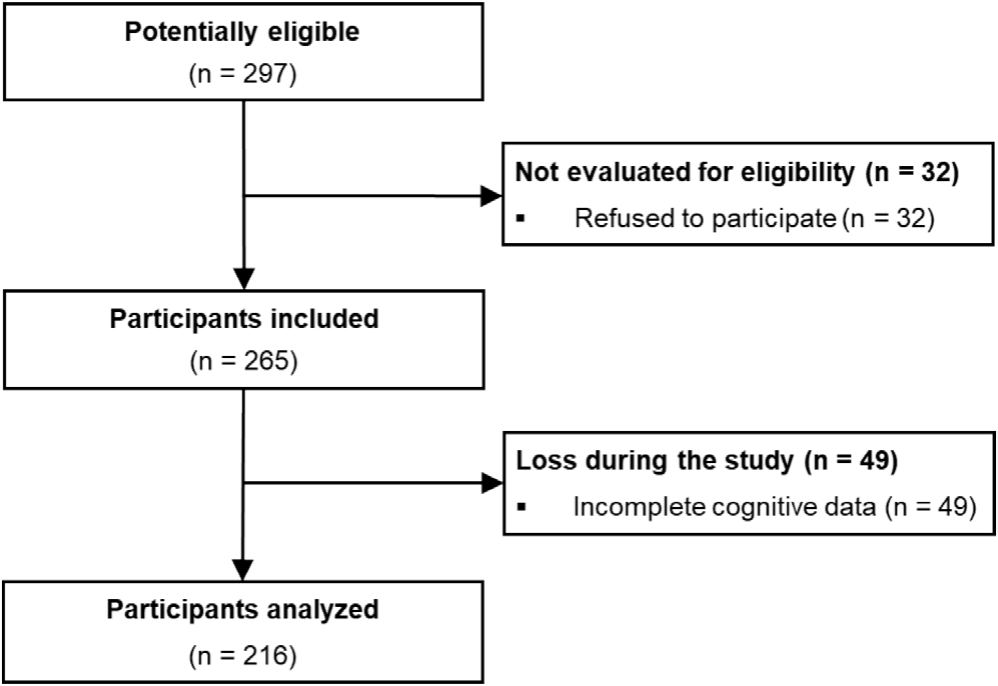
Sample flowchart.

The study included participants enrolled in high school‐integrated technical courses at the Federal Institute of Education, Science, and Technology of Paraíba (Instituto Federal de Educação, Ciência e Tecnologia da Paraíba—IFPB)—Sousa campus. The recruitment process included reaching out through social media and in classroom settings. To participate, individuals had to meet the criteria of being between the ages of 14 and 19, not having any psychological, psychiatric, or cognitive disorders, having a personal smartphone with the “digital wellbeing” function or a similar feature activated, and being regularly enrolled at the institution. Criteria for exclusion included voluntary withdrawal or lack of cognitive data. At the time of data collection, 297 students enrolled at the institution met the eligible criteria for participation. Of these, 32 refused to participate. Initially, 265 participants (94 boys, 171 girls) were included in the study. However, 49 participants (21 boys, 28 girls) were excluded due to incomplete cognitive data. Thus, the final sample consisted of 216 participants (73 boys, 143 girls) who were analyzed (see Figure 1).

### 2.3 Procedures

The study was conducted in the physical assessment laboratory of the Department of Physical Education at IFPB, with the ambient temperature controlled at approximately 24°C. Additionally, a covered sports court was utilized for physical testing. Data collection took place in the morning, from 7:30 to 11:00 AM, and was conducted by trained researchers. The study was divided into two distinct phases. In the first phase, participants completed questionnaires in person, directly interacting with the evaluator. Subsequently, with the participant's consent, the evaluator manually checked the screen time on the participant's smartphone to obtain daily usage data from the past week. Finally, participants were led to a separate, climate‐controlled room, free from visual distractions and external noise, to complete the Flanker test using a notebook. In the second phase, anthropometric measurements, muscle strength tests, and aerobic capacity assessments were performed.

### 2.4 24‐h Movement Behavior

#### 2.4.1 Moderate‐to‐Vigorous Physical Activity

The level of moderate‐to‐vigorous physical activity (MVPA) was assessed using the Global School‐based Student Health Survey (GSHS) (World Health Organization 2021). Weekly frequency and the duration (in minutes) of MVPA over a typical week were recorded. The MVPA level was categorized as either “insufficiently physically active” (< 60 min/day) or “physically active” (≥ 60 min/day) (World Health Organization 2021).

#### 2.4.2 Smartphone Screen Time

Screen time on smartphones was measured directly from the participant's device using a feature known as “digital wellbeing” or a similar tool. This feature tracks the daily usage time (in minutes) of the device. Data on screen time were collected for all 7 days of the previous week, from Monday to Sunday. Participants were instructed to access the app on their devices and provide the necessary data to the researcher, without any direct intervention by the researcher in manipulating the devices. The average daily screen time was then calculated from the recorded data and converted to hours per day.

#### 2.4.3 Sleep Duration

Sleep duration was measured using the GSHS (World Health Organization 2021). Sleep duration was recorded for a typical weekday (Monday through Friday) and for the weekend (Saturday and Sunday), followed by a weighted average calculation: (five × weekdays' sleep) + (two × weekend sleep) divided by 7 days. Sleep duration was categorized as inadequate if it was less than 8 h or more than 10 h per day for individuals under 18 years old and less than 7 h or more than 9 h per day for those 18 years or older. Sleep duration was considered adequate if it fell within these ranges according to age (Ross et al. 2020; Tremblay et al. 2016).

#### 2.4.4 Excessive Sleepiness

Daytime sleepiness was assessed using the Pediatric Daytime Sleepiness Scale (PDSS). This scale is validated for Brazilian children and adolescents (Felden et al. 2016; Ferrari Junior et al. 2018). The scale consists of eight multiple‐choice questions with a Likert‐type response format. At the end, scores are summed, and the total score ranges from zero to 32 points. Higher scores show greater levels of daytime sleepiness. A cutoff score of 15 points is used to identify excessive daytime sleepiness (Meyer et al. 2018).

### 2.5 Health‐Related Physical Fitness

#### 2.5.1 Aerobic Capacity

Aerobic capacity was assessed using the PACER test (The Cooper Institute 2013). In this test, the participant runs back and forth across a 20‐m space, reversing direction at each end and following a pace set by audio signals that progressively increase in speed. The test is stopped when the participant does not reach the line before the beep on two occasions (not necessarily consecutive) or voluntarily chooses to stop. The final score is the total number of laps completed, with each lap corresponding to 20 m. Maximum oxygen consumption (VO_2_max) in mL/kg/min was estimated using a validated equation for adolescents (Mahar et al. 2018).

#### 2.5.2 Muscle Strength

Muscle strength was assessed using the FitnessGram floor push‐up test (The Cooper Institute 2013), which requires participants to perform as many 90° push‐ups as possible at a constant pace. The test was conducted on a mat, with instructions provided by a pre‐recorded audio showing a cadence of 20 push‐ups per minute. The test was stopped if there was a second instance of improper form, voluntary exhaustion, or signs of extreme discomfort. The final score corresponded to the total number of correctly performed push‐ups.

#### 2.5.3 Excess Weight

Weight and height were measured using a digital scale (model W200, Welmy, Brazil) and a portable stadiometer (model ES2060, Sanny, Brazil), respectively. Body mass index (BMI) was calculated and converted to z‐score of each participant based on sex and age‐specific reference values from the World Health Organization (WHO Multicentre Growth Reference Study Group 2006). Participants were classified as having either normal weight or excess weight (overweight or obese).

### 2.6 Inhibitory Control

Inhibitory control performance was assessed using the modified Flanker task (Eriksen 1995). The assessment was conducted in an isolated room, free of external sounds and noises, with only a table, a chair, and a notebook. Participants positioned their dominant hand on the notebook, with their arms resting on the table. The software used to apply the modified version of the Flanker task was E‐Prime v3.0 (Psychological Software Inc.) (Walk et al. 2017). The test consists of fish images generated by software, representing combinations of congruent trials, in which the flanking fish at the ends follow the same direction as the target fish, and incongruent trials, where the flanking fish move in the opposite direction to the target or central fish. The study protocol included four phases. The first phase involved familiarization with 50 trials, followed by three additional phases, each consisting of 120 trials, with 40 trials per condition (congruent and incongruent). Each phase presented five yellow fish, 2.5 cm in size, on a blue background for 200 ms. Participants had response intervals of 1550, 1750, or 1950 ms, corresponding to interstimulus intervals of 1600, 1800, or 2000 ms, respectively. The results were recorded as reaction time (RT; in milliseconds) and accuracy (in percentage of correct responses) for both congruent and incongruent phases. For this study, only the RT from both the congruent and incongruent phases was used, with near‐perfect accuracy and low variability (congruent = 99.0 ± 1.7; incongruent = 98.6 ± 2.1).

### 2.7 Sociodemographic Variables

Age (years), sex (male and female), ethnicity (brown/black and white/yellow), and socioeconomic status (low and medium/high class) were collected using the GSHS (World Health Organization 2021) and by the Brazil Economic Classification Criterion 2022 (Brazilian Association of Research Companies 2022). Socioeconomic status was decided based on the possession of goods, housing conditions, and the education level of the head of household. Participants were initially categorized into three strata: low (0–22 points), medium (23–37 points), and high (38–100 points). For the purposes of network analysis, this variable was dichotomized into “low” (originally low stratum) and “medium/high” (a combination of the medium and high strata). The pubertal stage was estimated by peak height velocity (PHV) using an equation based on anthropometric data (Mirwald et al. 2002) and classified as pre‐pubescent (PHV −4 to −2), pubescent (PHV −1 to +1), and post‐pubertal (PHV ≥ +2). The variables included in the equation were lower limb length, trunk–brain height, age, weight, and height. Lower limb length and trunk–brain height were calculated by subtracting trunk–brain height from total height, measured with a portable stadiometer (model ES2060, Sanny, Brazil) while the participant was seated. Weight and height were measured using a digital scale (model W200, Welmy, Brazil) and a portable stadiometer (model ES2060, Sanny, Brazil), respectively.

### 2.8 Data Analysis

Continuous variables are presented as mean ± standard deviation, while categorical variables are described as absolute (n) and relative frequencies (%). A generalized gamma model with robust variance was applied to compare independent variables between sexes (SPSS software v.27, IBM Corp., Armonk, NY). A *p*‐value of < 0.05 was considered statistically significant. Network analysis was conducted to examine the interrelationship between 24‐h movement behaviors, health‐related physical fitness, and inhibitory control performance. This mathematical model is represented by nodes (variables) and edges (relationships between nodes). These relationships can vary in thickness, corresponding to the strength of associations, or in color, showing positive and negative correlations, highlighted by blue and red colors, respectively. We applied the “Fruchterman‐Reingold” algorithm to display the data in relative space, where variables with stronger associations are still close together, while those with weaker associations are repelled from each other. To enhance network accuracy, we employed the “Markov random field” model, which adds an “L1” penalty (regularized neighborhood regression). The regulation was estimated using the least absolute shrinkage and selection operator (Lasso), which controls the sparse network. The Extended Bayesian Information Criterion (EBIC) was used to select the Lambda regularization parameter, with a hyperparameter (γ) set at 0.5, providing greater parsimony and specificity in network creation (Costantini et al. 2015; Foygel and Drton 2010). In the analysis, centrality metrics known as betweenness, closeness, expected influence, and strength were also calculated to quantify the importance of the nodes. The first metric is the node that acts as the shortest path between two other nodes, mediating the connection between them (Opsahl et al. 2010). Closeness indicates how close a node is, on average, to all the other nodes (Jones et al. 2021). On the other hand, strength refers to the node with the greatest weight in the relationship, while expected influence sums the weights of these relationships, distinguishing between positive and negative edges to identify the most influential nodes in the network (Robinaugh et al. 2016). In addition, the accuracy and metrics stability of the network were evaluated by a bootstrap of 1000 resamples, leading to 1000 new networks comparing to the initial model and strengthening the results found. The network analyses were performed in JASP version 0.18.1.0 (Amsterdam University, Netherlands).

## 3 Results

Table 1 presents the characteristics of the study participants. Of the participants, 66.2% were girls and 33.8% were boys. On the pubertal stage, 77.8% were pubescent and 22.2% were post‐pubertal. No participants were classified as pre‐pubescent. Approximately one‐third of the participants were overweight (31.9%). In addition, most were insufficiently physically active (71.8%), had inadequate sleep duration (71.8%), and reported excessive daytime sleepiness (75.0%). In addition, regarding sex differences, girls were younger and had a higher proportion of pubertal participants (*p* < 0.05). Girls also had a higher proportion in the lower socioeconomic class and physical inactivity, as well as more screen time on smartphones (*p* < 0.05). Boys showed better performance in aerobic capacity and muscular strength, as well as shorter RT in both congruent and incongruent phases (*p* < 0.05). On the other hand, girls showed better accuracy in both congruent and incongruent phases (*p* < 0.05).

**TABLE 1:** Participant characteristics according to sex.

Variables	Overall	Girls	Boys	*p*
*N*, %	216	143 (66.2)	73 (33.8)	
Age, years	16.7 ± 1.2	16.4 ± 1.1	17.1 ± 1.2	< 0.001
Pubescent	168 (77.8)	132 (92.3)	36 (49.3)	< 0.001
Post‐pubertal	48 (22.2)	11 (7.7)	37 (50.7)	
Brown/black	105 (48.6)	66 (46.2)	39 (53.4)	0.312
White/yellow	111 (51.4)	77 (53.8)	34 (46.6)	
Lower class	60 (27.8)	47 (32.9)	13 (17.8)	0.019
Middle/upper class	156 (72.2)	96 (67.1)	60 (82.2)	
Height, m	1.65 ± 0.09	1.61 ± 0.06	1.73 ± 0.07	< 0.001
Weight, kg	62.7 ± 13.5	60.7 ± 12.2	66.7 ± 15.0	0.002
BMI, kg/m^2^	23.0 ± 4.2	23.4 ± 4.3	22.2 ± 3.9	0.052
Normal weight	147 (68.1)	93 (65.0)	54 (74.0)	0.183
Overweight/obesity	69 (31.9)	50 (35.0)	19 (26.0)	
VO_2_máx, ml/kg/min	35.6 ± 5.9	32.9 ± 3.0	40.4 ± 6.5	< 0.001
Upper body strength, rep	5.5 ± 6.9	2.3 ± 3.9	11.4 ± 7.5	< 0.001
Smartphone ST, min/day	7.1 ± 2.0	7.4 ± 1.9	6.4 ± 1.8	0.001
Adequate sleep duration	61 (28.2)	35 (24.5)	26 (35.6)	0.085
Inadequate sleep duration	155 (71.8)	108 (75.5)	47 (64.4)	
Physically active	61 (28.2)	32 (22.4)	29 (39.7)	0.007
Insufficiently physically active	155 (71.8)	111 (77.6)	44 (60.3)	
Excessive daytime sleepiness	162 (75.0)	111 (77.6)	51 (69.9)	0.213
Congruent RT, ms	474 ± 103	493 ± 112	437 ± 69	< 0.001
Congruent accuracy, %	99.0 ± 1.7	99.2 ± 1.4	98.7 ± 2.1	0.039
Incongruent RT, ms	507 ± 113	531 ± 123	460 ± 71	< 0.001
Incongruent accuracy, %	98.6 ± 2.1	99.0 ± 1.5	97.8 ± 2.7	< 0.001

*Note:* The data are presented as mean ± standard deviation (SD) for continuous variables, while categorical data are expressed as absolute frequencies (*n*) and relative frequencies (%).

Abbreviations: BMI, body mass index; ms, milliseconds; RT, reaction time; ST, screen time.

**FIGURE 2:**
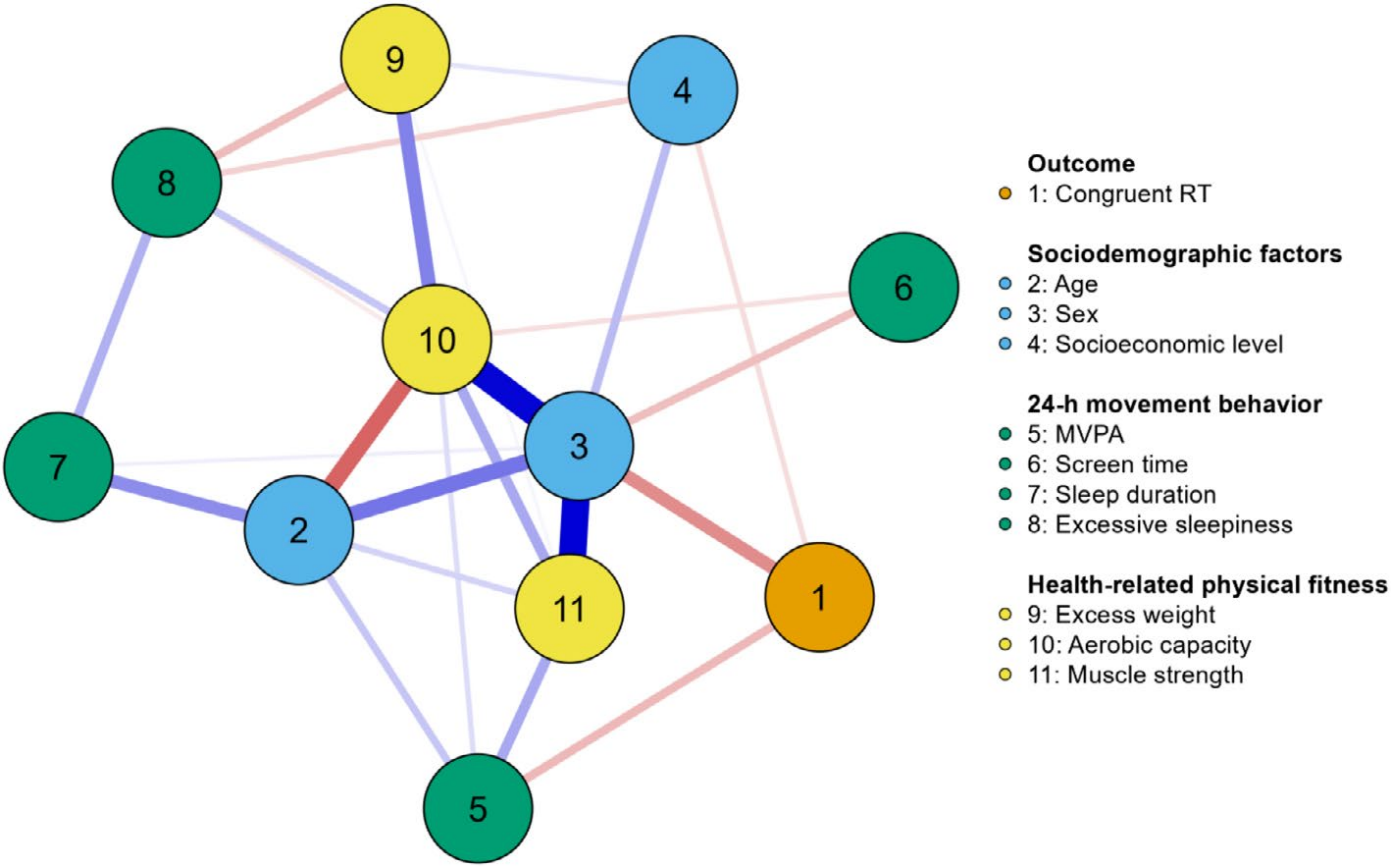
Network topology for the association between 24‐h movement behaviors, health‐related physical fitness, sociodemographic factors, and congruent reaction time in adolescents (*n* = 216). Blue edge: positive association; Red edge: negative association. 1 = Congruent reaction time (RT; milliseconds); 2 = Age (years); 3 = Sex (ref: female); 4 = Socioeconomic level (ref: lower class); 5 = Moderate to vigorous physical activity (MVPA; ref: insufficiently physically active); 6 = Smartphone screen time (hour/day); 7 = Sleep duration (ref: inadequate); 8 = Excessive sleepiness (ref: yes); 9 = Excess weight (ref: yes); 10 = Aerobic capacity (mL/kg/min); 11 = Muscular strength (repetitions).

**FIGURE 3:**
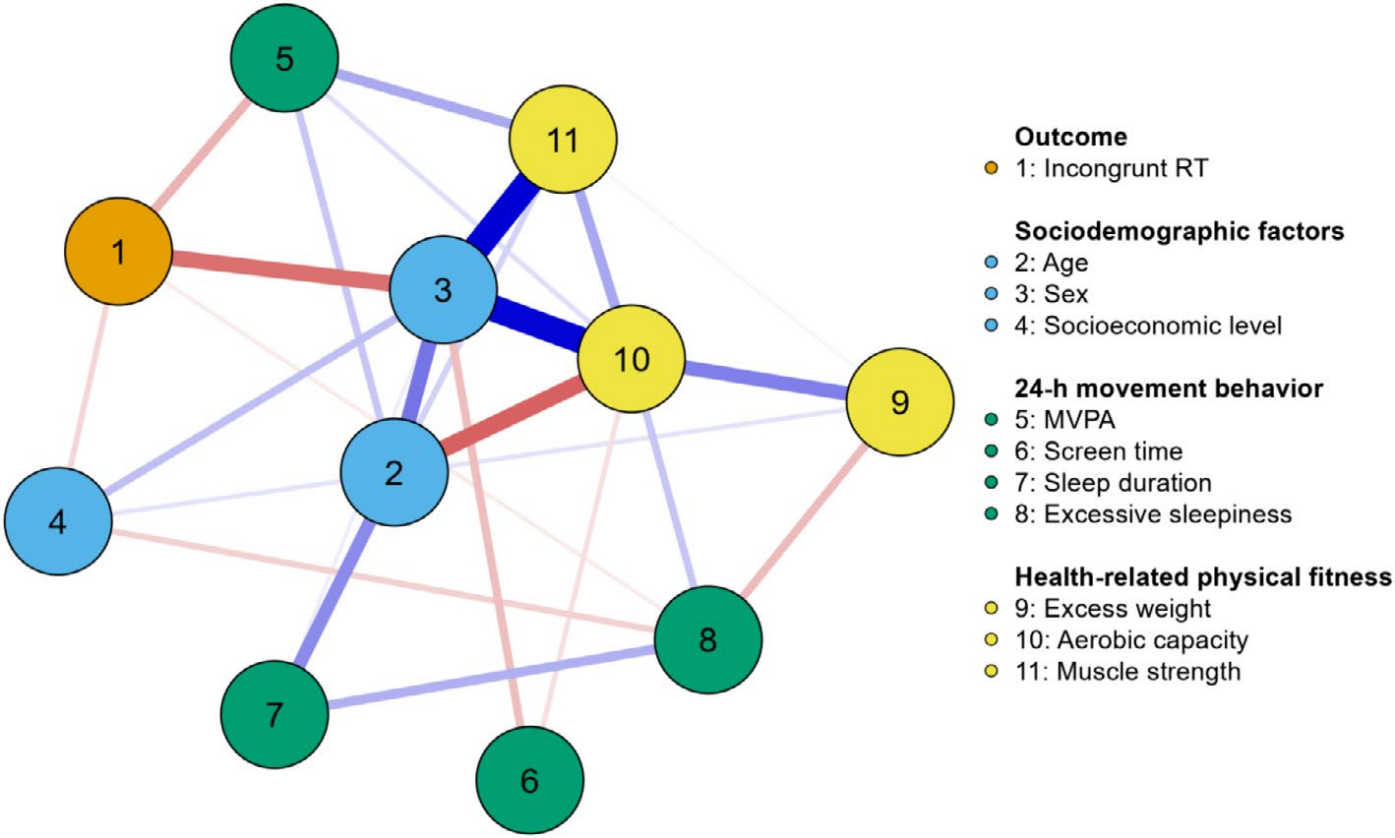
Network topology for the association between 24‐h movement behaviors, health‐related physical fitness, sociodemographic factors, and incongruent reaction time in adolescents (*n* = 216). Blue edge: positive association; Red edge: negative association. 1 = Incongruent reaction time (RT; milliseconds); 2 = Age (years); 3 = Sex (ref: female); 4 = Socioeconomic level (ref: lower class); 5 = Moderate to vigorous physical activity (MVPA; ref.: insufficiently physically active); 6 = Smartphone screen time (hour/day); 7 = Sleep duration (ref: inadequate); 8 = Excessive sleepiness (ref: yes); 9 = Excess weight (ref: yes); 10 = Aerobic capacity (mL/kg/min); 11 = Muscular strength (repetitions).

The results of the network analyses for models including congruent and incongruent phases are shown in Figures 2 and 3 (topology), Table 2 (weight matrix), and Table 3 (centrality measures). In the complex network of the congruent phase, sex (−0.190) and adherence to MVPA guidelines (−0.116) were directly associated with RT. Boys had lower RT than girls, and physically active participants had lower RT than insufficiently active peers. Additionally, the centrality measures highlight the importance of sex variables (betweenness: 2.660; closeness: 1.777; strength: 2.083; expected influence: 1.647), aerobic capacity (betweenness: 0.831; closeness: 1.352; strength: 1.432; expected influence: 0.879), and muscular strength (expected influence: 1.360) in the network topology.

**TABLE 2:** Association between 24‐h movement behaviors, health‐related physical fitness, and inhibitory control performance from a network analysis perspective in adolescents (*n* = 216).

Variable	1	2	3	4	5	6	7	8	9	10	11
Model 1											
Congruent RT (1)	—										
Age (2)	0.000	—									
Sex (3)	−0.190	0.229	—								
Socioeconomic level (4)	−0.057	0.000	0.113	—							
MVPA (5)	−0.116	0.095	0.000	0.000	—						
Smartphone screen time (6)	0.000	0.000	−0.108	0.000	0.000	—					
Sleep duration (7)	0.000	0.190	0.029	0.000	0.000	0.000	—				
Excessive sleepiness (8)	−0.037	0.000	0.000	−0.076	0.000	0.000	0.130	—			
Excess weight (9)	0.000	0.000	0.000	0.045	0.000	0.000	0.000	−0.111	—		
Aerobic capacity (10)	0.000	−0.259	0.417	0.000	0.057	−0.053	0.000	0.095	0.208	—	
Muscle strength (11)	0.000	0.073	0.425	0.000	0.143	0.000	0.002	0.000	0.014	0.143	—
Model 2											
Incongruent RT (1)	—										
Age (2)	0.000	—									
Sex (3)	−0.236	0.226	—								
Socioeconomic level (4)	−0.067	0.000	0.106	—							
MVPA (5)	−0.125	0.093	0.000	0.000	—						
Smartphone screen time (6)	0.000	0.000	−0.106	0.000	0.000	—					
Sleep duration (7)	0.000	0.190	0.029	0.000	0.000	0.000	—				
Excessive sleepiness (8)	−0.033	0.000	0.000	−0.076	0.000	0.000	0.130	—			
Excess weight (9)	0.000	0.000	0.000	0.045	0.000	0.000	0.000	−0.111	—		
Aerobic capacity (10)	0.000	−0.259	0.413	0.000	0.053	−0.053	0.000	0.094	0.209	—	
Muscle strength (11)	0.000	0.074	0.420	0.000	0.139	0.000	0.002	0.000	0.015	0.143	—

*Note:* 1 = Incongruent reaction time (milliseconds); 2 = Age (years); 3 = Sex (ref: female); 4 = Socioeconomic level (ref: lower class); 5 = Moderate to vigorous physical activity (ref: insufficiently physically active); 6 = Smartphone screen time (hour/day); 7 = Sleep duration (ref: inadequate); 8 = Excessive sleepiness (ref: yes); 9 = Excess weight (ref: yes); 10 = Aerobic capacity (mL/kg/min); 11 = Muscular strength (repetitions).

Abbreviations: MVPA, moderate to vigorous physical activity; RT: reaction time.

**TABLE 3:** Centrality measures per variable of the congruent and incongruent models in adolescents (*n* = 216).

Variable	Betweenness	Closeness	Strength	Expected influence
Model 1				
Congruent RT	−0.567	−0.181	−0.518	−1.633
Age	0.293	0.742	0.529	0.185
Sex	**2.660**	**1.777**	**2.083**	**1.647**
Socioeconomic level	−0.567	−0.889	−0.773	−0.574
MVPA	−0.567	−0.809	−0.493	−0.189
Smartphone screen time	−0.567	−1.105	−1.079	−1.037
Sleep duration	0.460	−0.520	−0.632	0.241
Excessive sleepiness	−0.567	−0.914	−0.402	−0.634
Excess weight	−0.567	−0.230	−0.567	−0.244
Aerobic capacity	**0.831**	**1.352**	**1.432**	**0.879**
Muscle strength	0.078	0.777	0.420	**1.360**
Model 2				
Incongruent RT	−0.567	0.099	−0.387	−1.749
Age	0.293	0.718	0.503	0.216
Sex	**2.660**	**1.746**	**2.124**	**1.528**
Socioeconomic level	−0.567	−0.973	−0.776	−0.578
MVPA	−0.567	−0.855	−0.506	−0.196
Smartphone screen time	−0.567	−1.123	−1.093	−0.996
Sleep duration	−0.460	−0.532	−0.645	0.280
Excessive sleepiness	−0.567	−0.913	−0.425	−0.587
Excess weight	−0.567	−0.240	−0.579	−0.205
Aerobic capacity	**0.831**	**1.326**	**1.396**	**0.902**
Muscle strength	0.078	0.746	0.388	**1.384**

Abbreviations: MVPA, moderate to vigorous physical activity; RT, reaction time.

In the complex network of the incongruent phase, the results were similar. Sex (−0.236) and adherence to MVPA guidelines (−0.125) were directly associated with RT in the incongruent phase. Specifically, boys showed lower RT than girls, and physically active participants had lower RT than insufficiently active peers. Furthermore, the centrality measures indicate the importance of the variables sex (betweenness: 2.660; closeness: 1.746; strength: 2.124; expected influence: 1.528), aerobic capacity (betweenness: 0.831; closeness: 1.326; strength: 1.396; expected influence: 0.902), and muscular strength (expected influence: 1.384) in the network topology.

## 4 Discussion

This study aimed to investigate the interrelationships between 24‐h movement behaviors, health‐related physical fitness, and inhibitory control performance in adolescents, using a complex network approach. The main findings were: (a) Adherence to MVPA recommendations was negatively associated with RT in both congruent and incongruent phases, indicating that physically active participants demonstrated better inhibitory control performance compared to their insufficiently active peers; (b) muscular strength showed a high expected influence in both network topologies; (c) centrality measures have shown that aerobic capacity plays a key role in mediating various connections (betweenness), being more centrally located, and consequently transmitting effects more quickly to other variables (closeness). Additionally, it demonstrates strong associations and is a highly influential variable in the network, though its influence is slightly lower than that observed for muscular strength; d) like aerobic capacity, sex was relevant in terms of betweenness, closeness, strength, and expected influence within the network topology. These findings may reflect sex differences in the study's main outcome.

The findings indicate that adherence to MVPA guidelines is negatively associated with RT in both the congruent and incongruent phases of the Flanker task. These results suggest that physically active adolescents exhibit better cognitive performance compared to their insufficiently active peers. These findings are consistent with previous studies using traditional analytical approaches, such as regression analysis (Westfall et al. 2018), and network analysis (Felin Fochesatto et al. 2023). In the network structure, MVPA appeared as a principal component directly associated with RT, reinforcing its relevance to inhibitory control performance. Additionally, MVPA showed strong interconnections with other key variables, such as aerobic capacity and muscular strength, highlighting its broader role in supporting both cognitive and physical health. Mechanistically, MVPA may enhance neuroplasticity by increasing cerebral blood flow, regulating neurotransmitters like dopamine and serotonin, and reducing stress (Li, Wang, et al. 2022; Li, Zhou, et al. 2022; Pedersen 2019). It is also essential for the development and maintenance of executive functions in adolescence (Diamond 2020; Heilmann et al. 2022). Moreover, MVPA contributes to brain health by increasing hippocampal volume, improving white matter integrity, enhancing brain activity efficiency, and reducing symptoms of anxiety and depression, in addition to promoting academic achievement (Erickson et al. 2019; Li, Wang, et al. 2022; Ruiz‐Ariza et al. 2017). These findings underscore the importance of meeting MVPA guidelines to support adolescents' cognitive and overall health.

The analysis revealed muscular strength as a highly influential node, both in the model that includes the congruent phase and in the incongruent phase. Muscular strength is a significant component of health‐related physical fitness and is associated with a range of physical and mental health benefits for adolescents (Bermejo‐Cantarero et al. 2021; Ruiz et al. 2006). Furthermore, several studies have demonstrated a positive association between muscular strength and inhibitory control (Contreras‐Osorio et al. 2022; Li, Zhou, et al. 2022; Solis‐Urra et al. 2021). Our findings have significant practical implications, as variables with expected influence values play a mediating role in activation, persistence, and network remission. This suggests that changes in the activation of the highly influential node are associated with alterations in the activation of other variables within the network (Robinaugh et al. 2016). The relationship between muscular strength and cognitive functioning in adolescents may be mediated by several factors, including the release of myokines, improvements in body composition, enhanced sensorimotor integration, and reduced fatigue (Muntaner‐Mas et al. 2022). These combined mechanisms may contribute to the positive impact of muscular strength on cognitive performance (Muntaner‐Mas et al. 2022). Thus, our findings emphasize the importance of the interconnection between muscular strength and not only physical health aspects but also inhibitory control performance in the adolescent population.

The results indicate that aerobic capacity has high values in all centrality measures, especially closeness, strength, and betweenness. These results suggest that aerobic capacity can generate positive changes in the observed network patterns, highlighting the importance of the position of this node, which acts as a hub by connecting other variables and, consequently, hyper‐connecting the system (Jones et al. 2021; Robinaugh et al. 2016). The high closeness centrality indicates that aerobic capacity is well positioned to efficiently influence or be influenced by other nodes within the network, underscoring its role in quickly disseminating effects throughout the system. Additionally, the strong centrality suggests that aerobic capacity has robust and numerous connections with other variables, further emphasizing its integral role in maintaining the network's overall coherence and stability. Previous studies have demonstrated both direct and indirect relationships between aerobic capacity and inhibitory control performance in adolescents (Cabral et al. 2021; Nieto‐López et al. 2020; Núñez et al. 2019; Westfall et al. 2018). Moreover, aerobic capacity, which directly reflects the overall effectiveness of the cardiovascular and respiratory systems in supporting physical activities, plays a crucial role in both physiological and psychological health (Belcher et al. 2021). Therefore, promoting physical activities that enhance aerobic capacity also stimulates the release of growth factors, such as brain‐derived neurotrophic factor and insulin‐like growth factor 1, which are essential for neuroplasticity (Diamond and Ling 2016; Ruiz‐Ariza et al. 2017). While the findings do not show this directly, engaging in physical activity, particularly MVPA, can enhance aerobic capacity, cardiovascular health, brain health, and cognitive functions (Cabral et al. 2021; Hillman et al. 2008). Therefore, our findings suggest an indirect relationship between aerobic capacity and inhibitory control performance in adolescents, indicating that better aerobic capacity may contribute to improved inhibitory control performance.

Sex emerged as the most central node in both networks, underscoring its organizing role in the interplay between movement behaviors, physical fitness, and cognition. This centrality likely reflects complex, multilevel sex differences: males exhibited superior physical fitness and cognitive performance, possibly due to neurobiological advantages, such as testosterone‐driven prefrontal maturation (Kurth et al. 2021), and the effects of accelerated pubertal development on executive function (Ojha et al. 2022). In contrast, females reported greater engagement in risk behaviors such as physical inactivity and excessive screen time, patterns consistent with global reports of gender disparities in physical activity (Guthold et al. 2020), which may stem from socioenvironmental barriers disproportionately affecting adolescent girls (e.g., limited access to sports, safety concerns; Duffey et al. 2021). Notably, these behavioral patterns were more strongly associated with negative outcomes among females, suggesting a differential vulnerability to environmental influences, as described in social–cognitive frameworks (Tompson et al. 2020). While biological sex is immutable, the network topology highlights modifiable behavioral targets: school‐based strategies aimed at overcoming barriers to physical activity in girls could help reduce observed disparities, and screen time reduction policies may yield benefits across sexes. Future studies should account for pubertal stage to better disentangle sex‐related effects from maturational confounders.

This study presents strengths and limitations. Firstly, it is innovative to apply a network analysis approach to establish the interconnections among various important variables in the context of adolescence, such as health risk behaviors, health‐related physical fitness, and inhibitory control. The employment of well‐established and recommended physical assessments by FitnessGram, along with the use of E‐Prime software to administer the Flanker task, ensures the consistency and reliability of the findings. Furthermore, the inclusion of a diverse sample of Brazilian adolescents from a federal public school enhances the external validity of the results, extending their relevance to similar demographic contexts. However, the study has some limitations. The measurement of physical activity and sleep, which was self‐reported through questionnaires, may have been subject to response biases. Furthermore, the relatively small sample size, particularly among male participants, may have limited the possibility of conducting more complex stratified network analyses and may also affect the generalizability of the findings to broader populations. Future studies should consider using more objective measurement methods, such as accelerometers and sleep monitoring devices, as well as increasing the sample size to improve the robustness and generalizability of the results.

The findings of this study have practical implications within the school setting, highlighting the necessity of promoting MVPA among adolescents. Adherence to MVPA guidelines can significantly enhance health‐related physical fitness and inhibitory control performance. Regular participation in MVPA is well documented to improve overall physical fitness, including aerobic capacity and muscular strength (Syväoja et al. 2014), and has been associated with improvements in cognitive performance (Fung et al. 2023). Therefore, incorporating regular measurements of muscle strength and aerobic capacity into school health programs may be crucial for monitoring and encouraging the physical and cognitive development of adolescents. These components in the complex network of variables suggest that muscle strength and aerobic capacity play central roles in interactions with inhibitory control. Furthermore, the identification of sex as a significant variable in the complex network indicates that intervention strategies should consider sex differences, addressing the specific needs of both boys and girls. For future research, we recommend longitudinal studies using the complex network approach to explore targeted interventions that may benefit adolescents' cognitive performance and general health.

## 5 Conclusion

Our findings demonstrate that adherence to MVPA guidelines is directly associated with better inhibitory control performance in adolescents. Physically active adolescents showed shorter response times in both congruent and incongruent phases of the Flanker task compared to their insufficiently active peers, indicating a direct benefit of physical activity for cognitive function. Network analysis further revealed that sex and health‐related physical fitness, particularly aerobic capacity and muscular strength, held central positions in the network structure, underscoring their indirect relevance to inhibitory control and their potential as targets for future interventions.

## Author Contributions

**R.S.C.:** conceptualization; methodology; writing – original draft preparation. **A.C.C.N.:** conceptualization; formal analysis; writing – original draft preparation. **J.P.R.S.:** investigation; data curation; project administration; writing – review and editing. **V.D.O.T.:** data curation; methodology; writing – review and editing. **I.A.R.:** funding; software; methodology; resources; writing – review and editing. **A.I.F.:** investigation; writing – review and editing. **P.F.R.B.:** formal analysis; writing – review and editing. **C.M.L.M.:** methodology; writing – review and editing. **R.A.V.B.:** investigation; formal analysis; conceptualization; data curation; funding acquisition; supervision; writing – original draft preparation.

## Conflicts of Interest

The authors declare no conflicts of interest.

## Data Availability

The data that support the findings of this study are available from the corresponding author upon reasonable request.
